# V3 midface lifting through a subciliary approach: a three-vector physiologic repositioning technique for midface rejuvenation, lower eyelid support, and lower lid-malar contour improvement

**DOI:** 10.3389/fsurg.2026.1910831

**Published:** 2026-09-11

**Authors:** Anyun Chen, Jianfeng Wang, Kang Liu, Fan Zhang, Jiaheng Xie

**Affiliations:** 1 Department of Plastic and Aesthetic, Xiangyang Huamei Medical Beauty Hospital, Xiangyang, China; 2 Department of Wound Repair and Plastic Burn, Hanchuan People’s Hospital, Hanchuan, China; 3 Department of Plastic and Aesthetic, Zeshang Xihe Medical Beauty Hospital, Wuhan, China; 4 Department of Medicine, South Asia University, Bishkek, Kyrgyzstan; 5 Department of Plastic Surgery, Xiangya Hospital, Central South University, Changsha, China

**Keywords:** lower blepharoplasty, malar fat pad, midface lift, orbicularis suspension, subciliary approach, tear trough

## Abstract

**Background:**

Conventional midface rejuvenation through temporal or preauricular incisions often fails to adequately restore the central midface and tear trough vector. We describe the “V3 midface lift"—a transcutaneous subciliary technique utilizing a three-vector physiologic repositioning system to address midface ptosis, lower eyelid bags, and lower lid-malar contour disharmony.

**Methods:**

A consecutive cohort of 42 patients (38 females, 4 males; mean age: 46.2 ± 6.8 years) who underwent the V3 midface lift between January 2022 and December 2024 was retrospectively analyzed. The technique incorporates an extended subciliary incision, selective orbicularis oculi release, conservative septal fat management, and multi-point periosteal/fascial suspension anchored along three specific vectors: the medial canthal line, lateral canthal line, and the inferior boundary of the malar fat pad (adjacent to the melolabial fold). Quantitative outcomes were assessed using pre- and postoperative lower lid-malar distance (LMD), tear trough rating scales, and the Global Aesthetic Improvement Scale (GAIS).

**Results:**

All 42 patients completed follow-ups ranging from 12 to 36 months (mean: 22.4 months). Significant reductions in lower lid-malar distance (mean reduction: 3.4 ± 0.8 mm, *P* < 0.001) and marked smoothing of the tear trough/malar groove were achieved. High patient satisfaction was recorded at 12 months (92.8% rated as “Very Much Improved” or “Much Improved” on GAIS). Postoperative complications were limited to transient chemosis (*n* = 3, 7.1%) and minor prolonged edema (*n* = 2, 4.8%), both resolving without secondary intervention. No permanent ectropion, hematoma, or facial nerve injury occurred.

**Conclusions:**

The V3 midface lift provides safe, stable, and durable midface elevation and lower lid-malar continuity through a minimalist subciliary access, representing an efficient option for central facial rejuvenation.

## Introduction

Aging of the central face involves a complex interplay of skin laxity, orbicularis oculi attenuation, orbital fat pseudoherniation, deep fat compartment atrophy, and downward migration of the malar fat pad (1, 2). These structural shifts disrupt the smooth contour connecting the lower eyelid to the cheek, creating a distinct tear trough deformity, accentuation of the lid-cheek junction, and flattening of the anterior malar eminence (3).

Traditional rhytidectomy procedures primarily deliver lateral traction, which frequently leaves central midface hollows and tear trough deformities under-corrected (4). Conversely, deep-plane or endoscopic midface lifts offer powerful repositioning but carry longer learning curves, higher risk to facial nerve branches, and prolonged recovery times (5). Extended transcutaneous lower blepharoplasty techniques have addressed these limitations, yet maintaining secure, multi-vector vertical suspension without increasing lower lid downward tension remains a surgical challenge (6).

To achieve direct, physiologic restoration of the lower lid-malar complex, we developed a three-vector suspension technique performed through a subciliary approach: the V3 midface lift. This method targets the superficial skin envelope, orbicularis oculi muscle, deep fascia, and descended malar fat pad simultaneously. In this report, we outline the surgical concepts, operative steps, and a consecutive clinical series evaluating its quantitative efficacy and long-term durability.

### Patient demographics and clinical baseline

Between January 2022 and December 2024, a total of 42 consecutive patients (38 females [90.5%] and 4 males [9.5%]) underwent the V3 midface lift and were included in this study. The mean age of the cohort was 46.2 ± 6.8 years (range: 34 to 62 years). Primary procedures accounted for 88.1% of cases (*n* = 37), while 11.9% (*n* = 5) were revision surgeries. Concomitant upper blepharoplasty was performed in 12 patients (28.6%). All patients completed clinical evaluations with a mean follow-up duration of 22.4 ± 7.2 months (range: 12 to 36 months).

### Inclusion Criteria

1. Moderate-to-severe central midface ptosis accompanied by lower eyelid bags and tear trough deformities (Barton Grade II or III).
2. Blunting or flattening of the anterior malar convexity with accentuation of the lid-cheek junction.
3. Minimal to moderate lower eyelid laxity suitable for subciliary myocutaneous suspension.

### Exclusion Criteria

1. Severe dry eye syndrome or pre-existing lower eyelid malposition (e.g., symptomatic ectropion or entropion).
2. Active systemic bleeding disorders or uncontrolled metabolic diseases.
3. History of extensive previous lower blepharoplasty with excessive tissue loss or orbicularis disruption.

### Standardized surgical sequence and anatomical workflow

**Figure 1:**
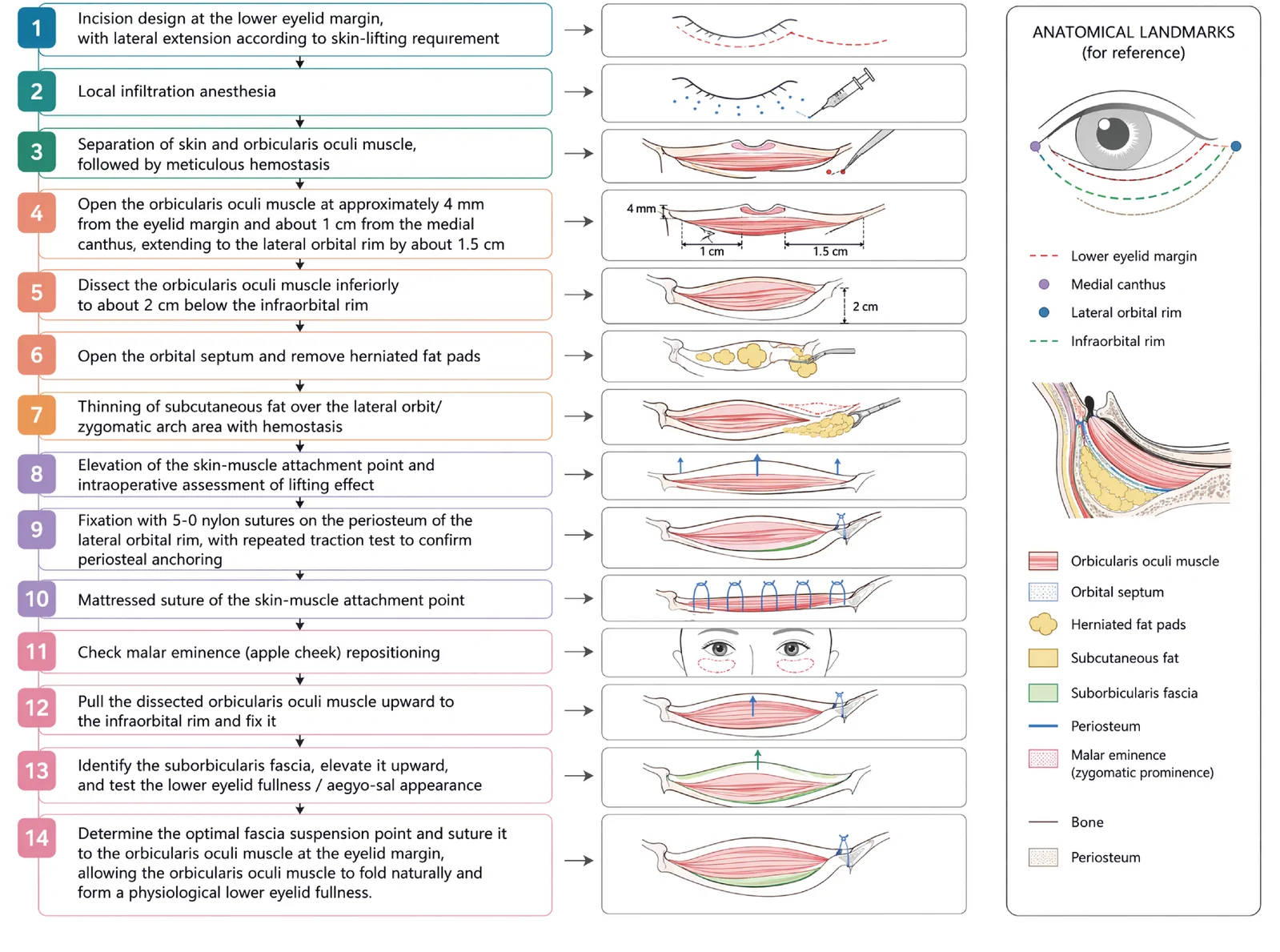
Step-by-step schematic surgical flowchart of the V3 midface lift procedure. The diagram illustrates the 14-step surgical sequence alongside corresponding anatomical illustrations and legends, detailing incision design, skin-muscle flap dissection, orbicularis release, septal fat management, multi-point vector elevation with periosteal anchoring at the lateral orbital rim, and superior resuspension of the orbicularis oculi and deep suborbicularis fascia to re-establish natural pretarsal roll (aegyo-sal contour) fullness.

The operational protocol for the V3 midface lift is hierarchically structured into a 14-step surgical sequence (Figure 1). Following local infiltration anesthesia along the subciliary line, a skin-muscle flap is dissected with meticulous release of the orbicularis retaining ligament (ORL) to approximately 2 cm below the infraorbital rim. After conservative management of herniated orbital fat and thinning of the lateral orbital subcutaneous fat pad, structural resuspension is executed. Multi-point periosteal fixation along the three primary vectors is confirmed via intraoperative traction testing using 5-0 nylon sutures. Crucially, the suborbicularis fascia is elevated superiorly and anchored to the pretarsal orbicularis muscle near the eyelid margin, promoting natural muscle folding to recreate physiologic pretarsal fullness (aegyo-sal contour) without creating tension on the lower eyelid margin (Figure 1).

### Surgical technique

#### 1. Preoperative vector mapping

**Figure 2:**
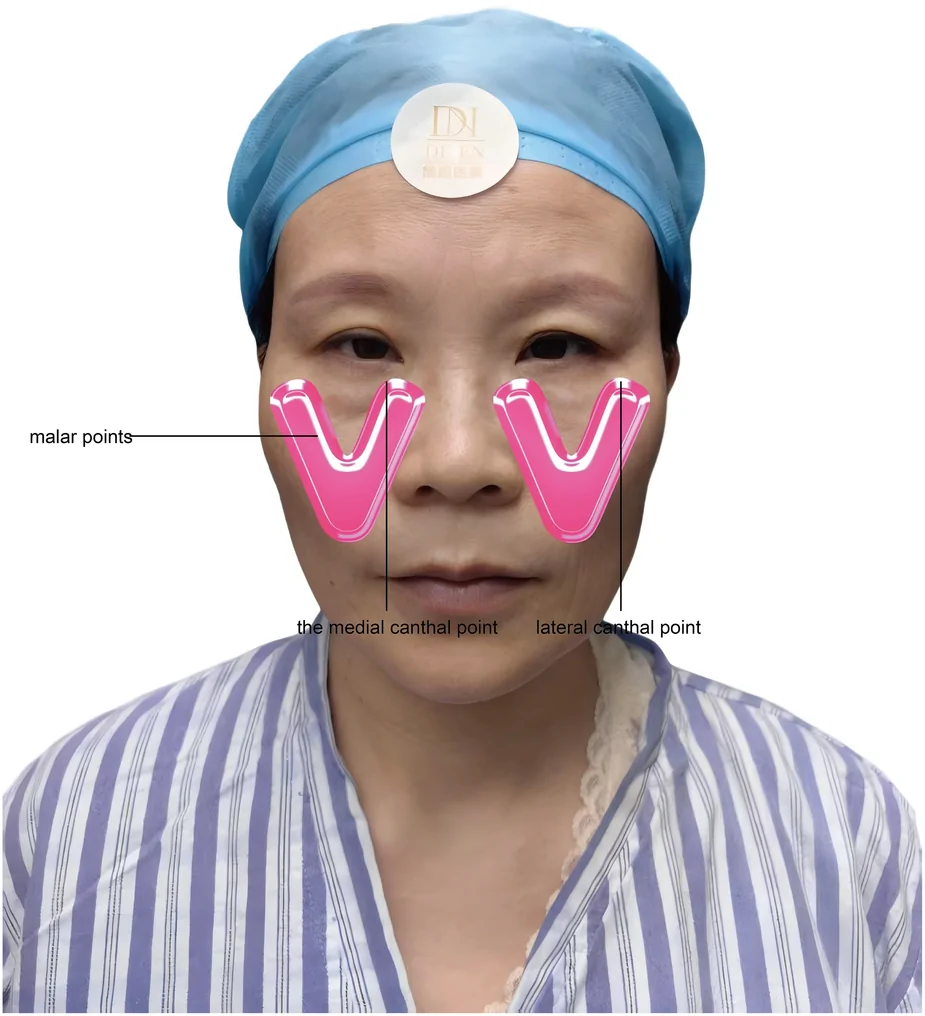
Preoperative design demonstrating the three-vector (V3) lifting system. Preoperative surface markings indicating the three principal vertical lifting vectors: Vector 1 (Medial Vector) extending from the medial canthal region down through the tear trough; Vector 2 (Lateral Vector) extending from the lateral canthus toward the zygomatic arch; and Vector 3 (Malar Vector) passing through the point of maximum malar projection down to the inferior boundary of the malar fat pad (adjacent to the melolabial groove).

With the patient in an upright sitting position, three key vertical vectors (V3) are marked on the midface (Figure 2):

- **Vector 1 (Medial Vector):** Extends vertically from the medial canthal region down through the tear trough toward the superior border of the nasolabial fold.
- **Vector 2 (Lateral Vector):** Extends from the lateral canthus toward the zygomatic arch.
- **Vector 3 (Malar Vector):** Passes through the point of maximum malar projection down to the inferior boundary of the malar fat pad (adjacent to the melolabial groove).

#### 2. Incision, dissection, and septal management

A subciliary incision is used and extended laterally beyond the orbital rim according to the amount of redundant skin requiring redraping. After local infiltration anesthesia, the skin is separated from the orbicularis oculi muscle. Meticulous hemostasis is obtained before proceeding deeper. Approximately 1 cm lateral to the medial canthus and 4 mm below the ciliary margin, the orbicularis is opened and the dissection is carried laterally toward the orbital rim. The orbicularis is then released inferiorly to approximately 2 cm below the infraorbital rim, releasing the orbicularis retaining ligament (ORL) and zygomaticocutaneous ligaments (ZCL) as needed to fully mobilize the malar soft tissue complex. The orbital septum is opened when indicated, and herniated fat is conservatively removed or transposed into the tear trough defect.

#### 3. Three-vector fixation and resuspension

The subcutaneous fat of the lateral orbital region (above the zygomatic arch) is judiciously thinned as needed. Tissue elevation is performed along the three pre-designed vectors:

- **Deep Anchoring:** The sub-orbicularis fascia and deep malar soft tissues along Vector 3 are recruited upward and secured to the lateral orbital periosteum using 5-0 clear nylon or PDS sutures.
- **Muscle Resuspension:** The lateral orbicularis oculi muscle flap (Vectors 1 and 2) is advanced superolaterally and anchored to the internal rim of the lateral orbital periosteum above the lateral canthal level.
- **Pretarsal Fullness Creation:** The deep fascial layer inferior to the orbicularis is anchored to the pretarsal orbicularis, allowing the muscle to fold naturally, restoring a physiologic pretarsal roll rather than an artificial bulge.

#### 4. Skin closure

Redundant skin is redraped tension-free in a superior-lateral direction. Excess skin (typically 2 to 4 mm) is trimmed conservatively. Skin closure is performed with 6-0 polypropylene sutures, which are removed on postoperative day 5 to 7.

#### Clinical results and quantitative outcomes

All 42 surgeries were completed without intraoperative complications. Patients were evaluated preoperatively and at 1, 6, 12, and 24 months postoperatively under standardized clinical studio conditions.

### Perioperative recovery and timeline assessment

**Figure 3:**
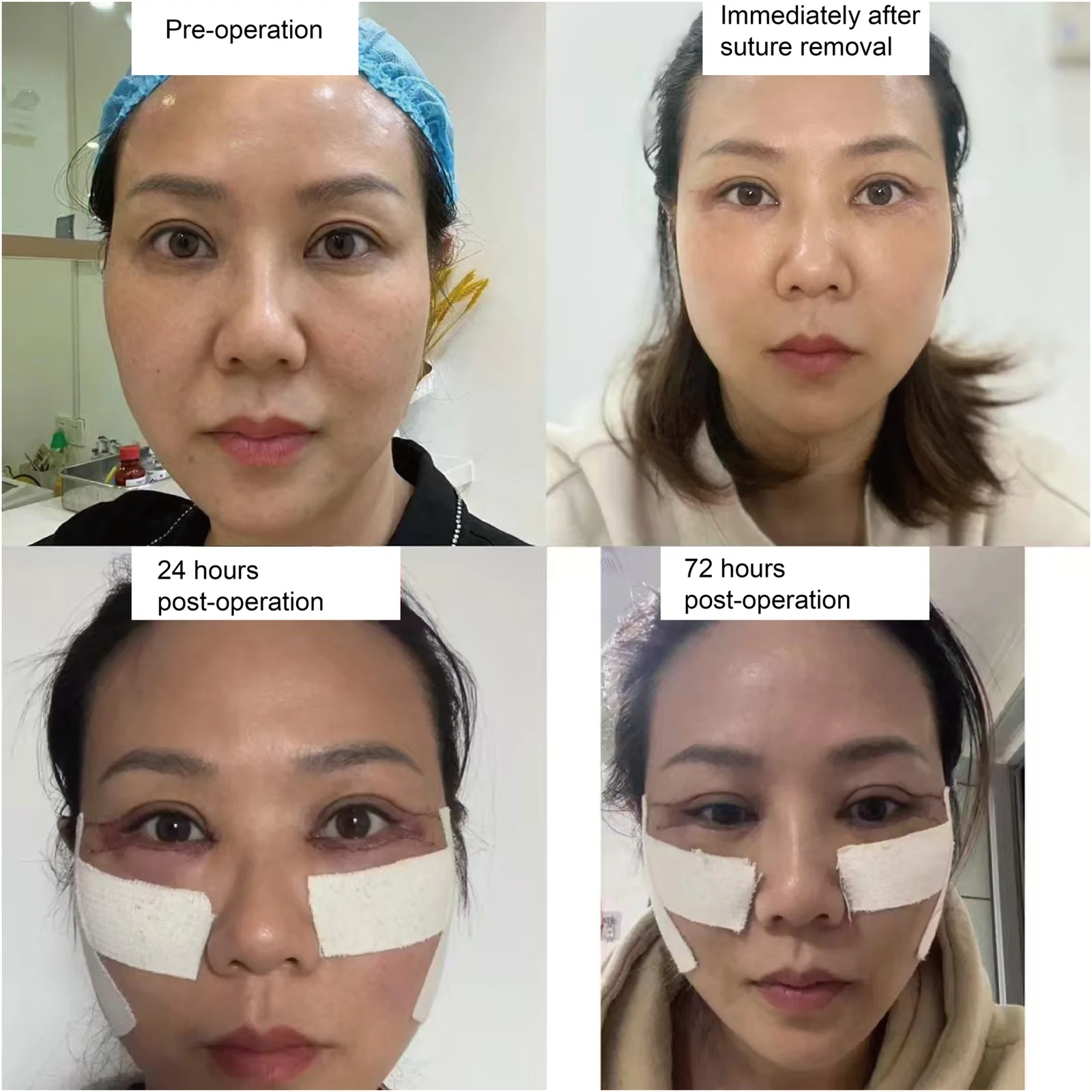
Representative perioperative recovery course of a patient undergoing the V3 midface lift. Sequential clinical photographs document the surgical trajectory at baseline (Pre-operation), 24 h post-operation (showing peak mild edema and supportive taping along vector tension lines), 72 h post-operation (demonstrating progressive swelling resolution), and immediately after suture removal (showing primary incision healing, minimal erythema, and early smoothing of the cheek-lid continuum).

To illustrate the acute recovery trajectory of the V3 midface lift, serial photographs were documented across the early perioperative phase. Mild to moderate subcutaneous edema and local swelling reached their peak at 24 h post-operation, accompanied by visible supportive steristrip taping over the midface vector anchoring regions. By 72 h post-operation, peri-incisional edema and tissue swelling had visibly subsided without significant ecchymosis or severe chemosis. Immediately following suture removal (postoperative day 5 to 7), the subciliary skin incision demonstrated excellent primary healing with minimal tension, significant improvement in tear trough contour, and early restoration of a smooth lower lid-malar junction. (Figure 3).

### Objective contour measurements

The distance between the pupil center and the lowest point of the lid-cheek junction (Lower Lid-Malar Distance, LMD) was measured using standardized medical photographs. The mean LMD decreased significantly from 18.6 ± 1.4 mm preoperatively to 15.2 ± 1.1 mm at 12 months postoperatively (*P* < 0.001), reflecting significant midface elevation and shortening of the lower eyelid vertical height.

### Representative case descriptions

**Figure 4:**
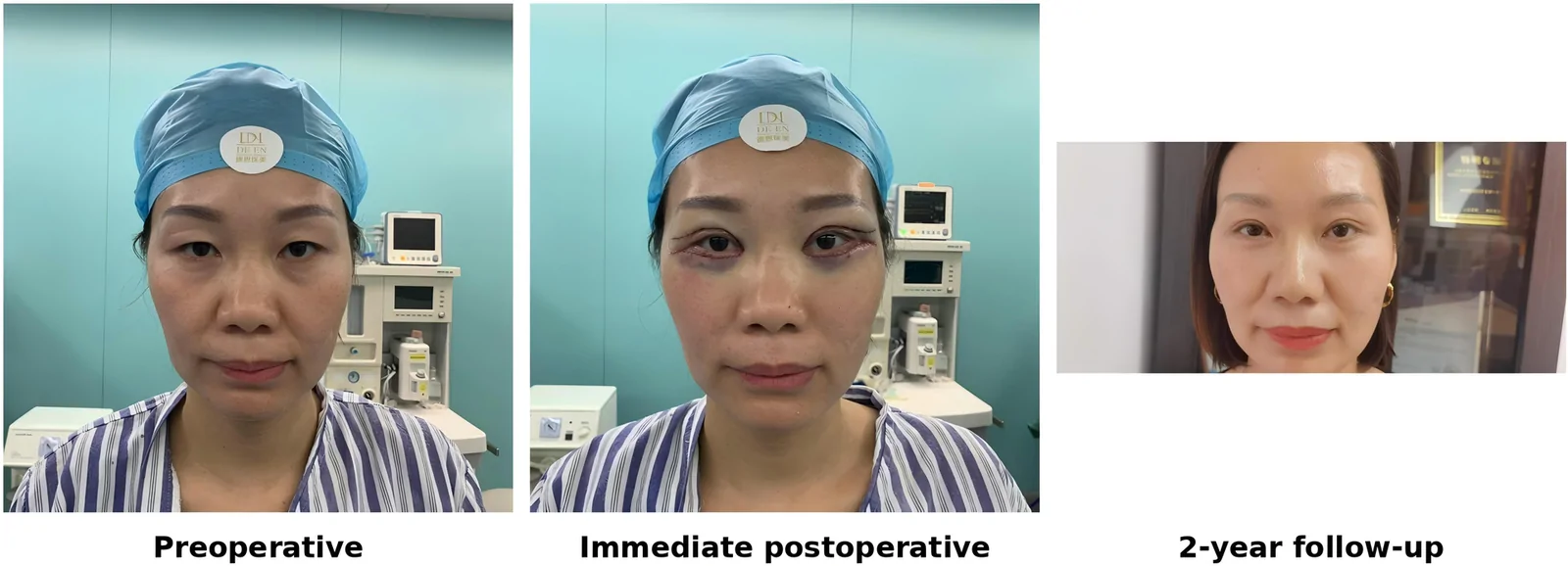
Representative case 1. Preoperative baseline, immediate postoperative appearance, and 2-year follow-up of a 45-year-old female, demonstrating sustained lower lid-malar continuity, elimination of infraorbital hollowing, and durable midface elevation.

**Figure 5:**
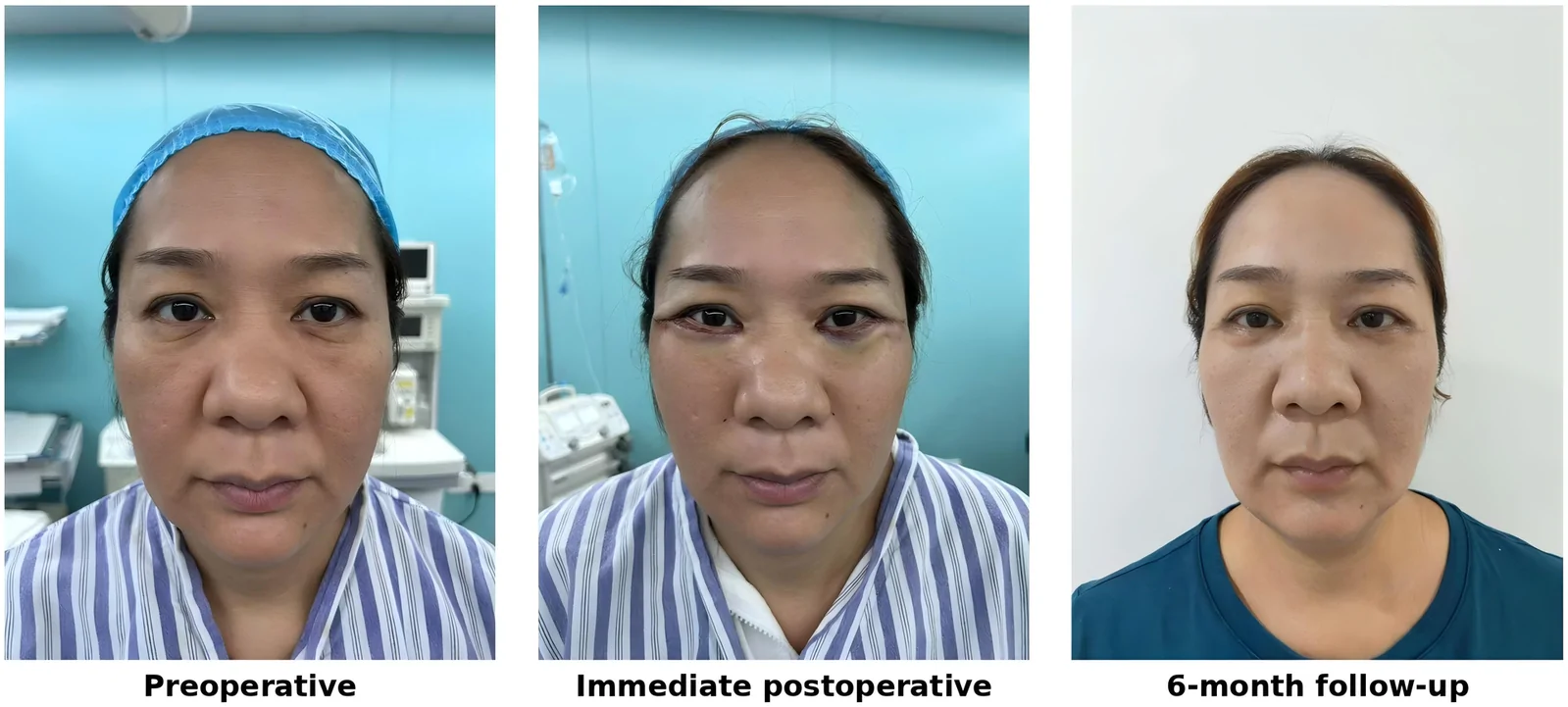
Representative case 2. Preoperative baseline, immediate postoperative appearance, and 6-month follow-up of a 48-year-old female, showing correction of orbital fat pseudoherniation, smoothing of the tear trough, and natural pretarsal roll restoration without scleral show or lid margin distortion.

**Figure 6:**
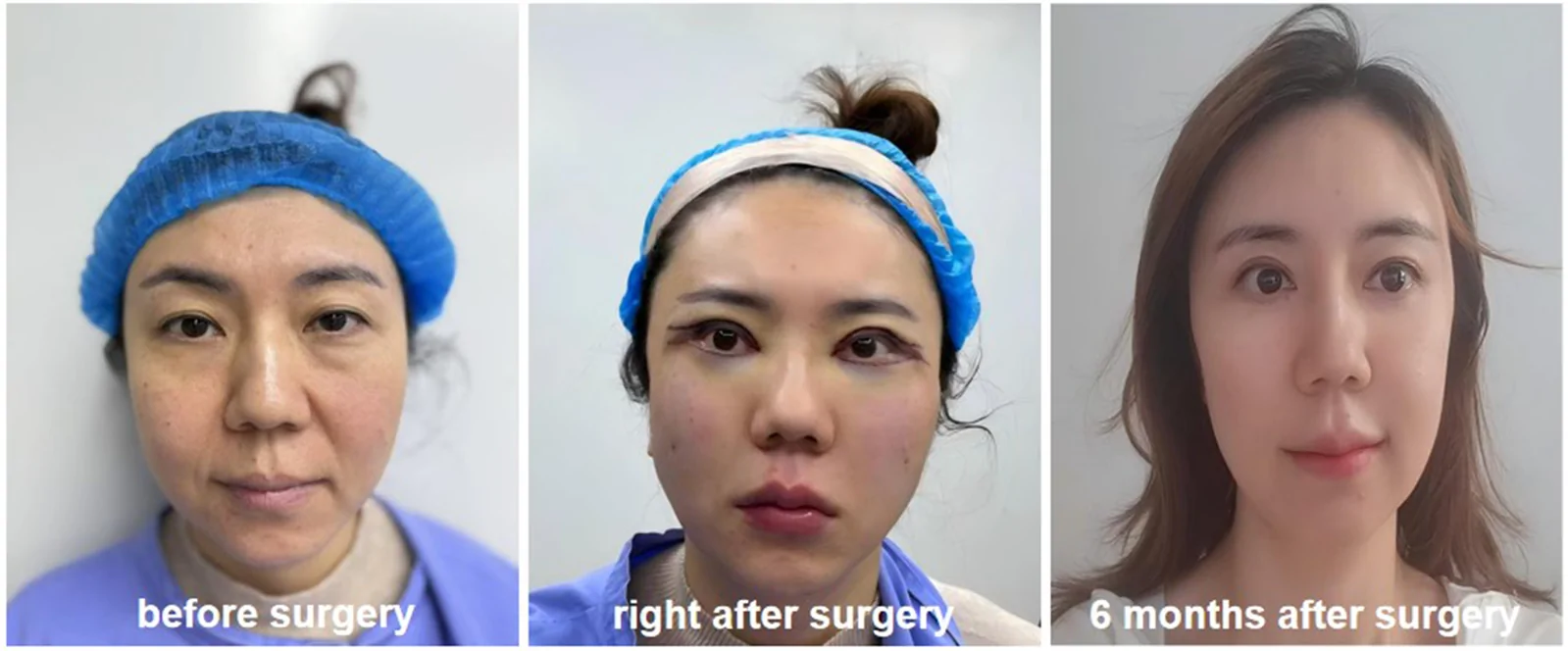
Representative case 3. Standardized clinical photographs of a 42-year-old female treated with the V3 midface lift: preoperative baseline (left), immediate postoperative appearance (middle), and 6-month postoperative follow-up (right). The outcome demonstrates stable midface elevation, seamless rejuvenation of the lid-cheek junction, complete resolution of tear trough grooving, and well-matured subciliary healing without residual deformity or eyelid malposition.

- Case 1 (Figure 4): A 45-year-old female presented with prominent tear trough deformities, midface soft-tissue ptosis, and flattening of the anterior malar eminence. Preoperative evaluation demonstrated significant disruption of the lower lid-malar transition. Immediate postoperative views confirmed effective vertical elevation of the malar complex and elimination of infraorbital hollowing. At the 2-year follow-up, a smooth, continuous cheek-lid junction was well-maintained with long-term stability and restored anterior malar projection.
- Case 2 (Figure 5): A 48-year-old female presented with moderate infraorbital fat pseudoherniation, deep tear trough accentuation, and central midface sagging. Following the V3 midface lift, immediate postoperative photos showed tension-free tissue repositioning and initial edema resolution. At the 6-month postoperative follow-up, the lower eyelid contour was significantly smoothed, orbital bags were fully corrected, and natural pretarsal fullness was established without scleral show or lower lid margin distortion.
- Case 3 (Figure 6): A 42-year-old female presented with pronounced tear trough depression, deep nasolabial folds, midface soft-tissue descent, and severe flattening of the anterior malar projection. Immediate postoperative photographs demonstrated significant structural repositioning of the descended midface fat pad, smooth effacement of the lid-cheek transition, and accurate tension-free closure along the subciliary margin. At the 6-month postoperative follow-up, the patient exhibited well-settled midface volume restoration, complete obliteration of the tear trough deformity, inconspicuous subciliary scarring without lateral canthal contracture or pigmentation changes, and natural preservation of the lower eyelid contour with no scleral show.

### Complication profile

No cases of permanent ectropion, retrobulbar hematoma, infection, or facial nerve injury were observed.

- **Transient Chemosis:** 3 cases (7.1%), resolved within 2 weeks with topical anti-inflammatory drops.
- **Prolonged Subcutaneous Edema:** 2 cases (4.8%), resolved by 6 weeks with warm compresses and physical therapy.
- **Minor Asymmetry:** 1 case (2.4%), required no secondary intervention as the patient was satisfied with the overall outcome.

### Patient satisfaction (GAIS score at 12 months)

- Very Much Improved (Grade 1): 28/42 (66.7%)
- Much Improved (Grade 2): 11/42 (26.2%)
- Improved (Grade 3): 3/42 (7.1%)
- Unchanged (Grade 4)/Worse (Grade 5): 0/42 (0%)

## Discussion

The V3 technique is conceptually distinct from lateral skin traction alone. Instead, it emphasizes direct restoration of the lower eyelid-malar unit through a subciliary route, with combined treatment of the superficial skin envelope, orbicularis oculi, deeper fascial layer, and descended malar fat (2, 3).

### Advantages of the three-vector system

Single-vector vertical or lateral traction often results in an unnatural “swept” appearance or recurrent midface sagging due to focal stress concentrations (7). The V3 system distributes lifting tension across three functional anatomical points:

1. **Medial Vector:** Corrects the tear trough and redrapes the thick skin-muscle layer over the inner infraorbital margin.
2. **Lateral Vector:** Re-establishes lateral orbital rim support and counteracts downward eyelid pull.
3. **Malar Vector (Inferior Boundary of the Malar Fat Pad):** Lifts the heavy descent of the midface from its true inferior margin near the melolabial fold, restoring youthful anterior malar projection.

### Comparison with classical techniques

Compared to classical procedures like Rohrich's multi-step lower blepharoplasty or subperiosteal midface lifts, the V3 technique offers key advantages (5, 6): (1) Lower neurological risk due to maintaining dissection in the pre-zygomatic sub-orbicularis plane above the periosteum, minimizing risk to the zygomatic and buccal branches of the facial nerve; (2) Natural aesthetic restoration by combining orbicularis flap advancement with deep fascial suspension to recreate a youthful pretarsal roll while smoothing the lid-cheek junction without creating a hollowed or skeletonized lower lid appearance (8).

### Limitations

1. **Retrospective Design:** Although data were collected consecutively, prospective randomized controlled studies comparing V3 with subperiosteal endoscopic midface lifts are warranted.
2. **Photographic Standardization:** Minor variations in ambient light and facial expression across multi-center follow-ups remain an inherent challenge in retrospective aesthetic studies, though strict calibration protocols were applied.

## Conclusion

The V3 midface lift is a reliable, three-vector subciliary technique that achieves physiologic repositioning of the midface soft tissue complex. By restoring lower eyelid support, smoothing the tear trough, and re-establishing malar convexity with low complication rates, this technique represents a practical and effective approach for central facial rejuvenation.

## Data Availability

The original contributions presented in the study are included in the article/Supplementary Material, further inquiries can be directed to the corresponding author.
